# A dual-process approach to prosocial behavior under COVID-19 uncertainty

**DOI:** 10.1371/journal.pone.0266050

**Published:** 2022-03-29

**Authors:** Daniela Costa, Nuno Fernandes, Joana Arantes, José Keating

**Affiliations:** Psychology Research Center, University of Minho, Braga, Portugal; Universitá Cattolica del Sacro Cuore, ITALY

## Abstract

Uncertainty has been shown to reduce the willingness to cooperate in various social dilemmas and negatively affect prosocial behavior. However, some studies showed that uncertainty does not always decrease prosocial behavior, depending on the type of uncertainty. More specifically, recent research has shown that prosocial behavior tends to increase under impact uncertainty—uncertainty about the consequences for others if they become infected. In addition, researchers have argued that intuition favors prosocial behavior while deliberation leads to selfish behavior. Our study explored how intuitive (time pressure) or deliberate mental processing, under outcome, or impact uncertainty affect prosocial behavior in the context of the COVID-19 pandemic. Our sample consists of 496 participants, and we used a 4 (COVID-19 scenario: Control vs. Impact Uncertainty vs. Worst-Case vs. Indirect Transmission) by 2 (decision time: time delay vs. time pressure) between-subjects design. Results suggest that participants are more inclined to stay at home (prosocial intention) when forced to make their decisions intuitively rather than deliberately. Additionally, we found that uncertainty does not always decrease prosocial behavior. It seems that uncertainty does not affect the prosocial intention in a scenario with a real infectious disease. These findings suggest that the distinction between outcome and impact uncertainty may be due to the realism of experimental stimuli interventions.

## Introduction

With the emergence of the new SARS-CoV-2 virus, also known as Coronavirus (COVID-19), we became more conscious of how our actions and decisions can impact others’ well-being. We make decisions that affect others in our everyday lives, and we can never be too certain about the impact of those decisions [1].

The COVID-19 pandemic is one of the most significant health threats of the last century. At the moment, there have been more than 200 million confirmed cases of COVID-19, including 4.5 million deaths worldwide reported to the World Health Organization (WHO). The high spread of the COVID-19 is partially explained by asymptomatic people who are often unaware of being infected but still transmit the virus [2–4]. For this reason, epidemiologists and health experts have recommended several measures to minimize the number of infections, including recommendations for people to stay at home [5]. At the beginning of the COVID-19 pandemic, many countries enforced lockdowns to slow Coronavirus spread. However, as case numbers diminished and the lockdowns lifted in May of 2020, many people believed the worst was surpassed and began socializing again without considering the protective measures. Although at the end of 2020 and the beginning of 2021, several countries had to implement additional curfews, stay-at-home orders, and other measures to fight the new peaks of infections and deaths. For example, in New Zealand, anticipatory measures, such as lockdown, and mandatory quarantine measures, helped combat the spread of COVID-19 [6]. Reinforcing that, if countries impose a near-total lockdown where the population has minimal movement and interpersonal contact for several weeks, they can quickly return to the "new normal" where it can be removed several COVID-19 restrictions. Removing restrictions does not mean returning to the prepandemic normal but instead deliberately transitioning to the "new normal" while being ready to reimpose measures if and when necessary [7]. For example, daily life returned to the "new normal" even before the vaccination started in Singapore and New Zealand [7, 8].

Because it is difficult to monitor every individual activity, it is critical to understand how to communicate these measures and encourage people toward adopting prosocial behaviors [9]. In particular, appeals, messages, and news can effectively promote the desired behavioral changes because they can reach a large audience through mass media like television and social media. However, when communicating these messages, policymakers, public health officials, and others should consider which messages effectively promote the acceptance of health measures against the Coronavirus [1, 10, 11]. Health communication with the public has become even more important since, according to the WHO, the recent upsurge in noncompliance with COVID-19 protective measures is likely to be a product of Pandemic Fatigue [12]. Pandemic Fatigue has been defined as a "demotivation to follow recommended protective behaviors, emerging gradually over time" [12].

Previous research in the field of health communication revealed that people are more risk-averse whenever their decisions affect others than themselves. So, focusing on *others’ risks* (rather than oneself) may be more effective in convincing individuals to practice public health behaviors [9]. In a recent study, the authors found that prosocial framing is a more compelling message than self-interested framing to encourage people to embrace COVID-19 preventive behaviors [11]. Similar to previous findings, another study found that informing subjects that the COVID-19 is a threat to "their community" increases intentions of wearing a face-covering mask [10]. However, in a replication study to the Japanese context, the authors found that all evaluated messages which emphasize the benefits of preventive behaviors for "*personal*", "*public*", "*personal and public*", or "*your family*" increased prevention intentions relative to a baseline [13].

Besides the importance of the messages conveyed, one should also consider the importance of the individual differences for the embracement of prosocial behavior in the COVID-19 pandemic. Previous research has examined the relationship between preventive behaviors and self-reported risk, risk-aversion, empathy, personality, and prosociality [14–20].

### Uncertainty and prosocial behavior

Infectious disease epidemics give rise to community uncertainty, as demonstrated by the recent public health emergencies, including SARS, H1N1, Ebola, and Zika [21]. Likewise, moral decision-making during a pandemic typically involves uncertainty [9]. There are several uncertainties during the pandemic of COVID-19, such as if we are contaminated and if we will infect others.

In experimental economic games, uncertainty has been shown to reduce the willingness to cooperate and behave prosocially [22–26]. The decreases in prosociality might occur due to people using the uncertainty about the social impact of their health behaviors as an excuse to adopt self-centered narratives [9, 27, 28] that allow them to maintain a positive self-image [1]. In addition, research suggests that people may be less willing to sacrifice for others’ benefits when they are uncertain [29, 30].

Although some studies tried to understand the negative effect of uncertainty on cooperation, recent research suggested that uncertainty does not always promote selfish behaviors [1]. In this study, the authors introduced a distinction between two types of uncertainty: outcome uncertainty (uncertainty about whether a decision will lead to a particular outcome) and impact uncertainty (uncertainty about how the negative outcome will impact others’ well-being).

In a series of 7 studies, similar to previous studies, the authors found decreased prosocial behavior under outcome uncertainty [1]. In contrast, prosocial behavior increased under impact uncertainty in incentivized economic decisions and hypothetical medical decisions concerning infectious disease threats. In their study regarding the hypothetical medical decision, participants read a hypothetical case of a contagious disease, and participants had to decide whether they would go to work while sick. Uncertainty was manipulated by varying the participants’ information about the vulnerability of people they might infect at work. Participants in the impact uncertainty and worst-case conditions were significantly more likely to stay home than participants in the control condition. This study suggested that focusing on impact uncertainty may encourage people to make sacrifices for others even if they are uncertain of the outcome.

Furthermore, this study used an implicit/explicit manipulation paradigm [31]. In this paradigm, participants made two possibility judgments (the possibility that co-workers are vulnerable; and the possibility to infect co-workers) presented either under time pressure (implicit condition) or without time limit (explicit condition). Following their possibility judgment, participants indicated whether they would go to work in the scenario they had read. In addition to the reported uncertainty effect, the authors also found that participants who made implicit possibility judgments (time pressure) were less likely to indicate they would go to work -prosocial behavior- than those who made explicit possibility judgments [1].

### The dual-process approach to social decisions

Similar to the previous result where participants in the time pressure condition were more likely to behave prosocially than participants in a time delay condition [1], research has been conducted in recent years to explore this relationship [32].

Experimental manipulations of decision time are interpreted within the framework of the dual-process approach, which conceptualizes decisions as arising from a competition between intuitive ("automatic") versus deliberative ("controlled") cognitive processes [33]. Research on dual-process assumes the existence of two types of process: Type 1 and Type 2 [32, 34–36]. Type 1 processing is autonomous and makes minimal demands on working memory resources [36]. In addition, Type 1 processes have other associated features as they tend to be fast, nonconscious, and not put a heavy load on central processing capacity, meaning they tend to be associative and automatic [33, 35, 36]. In this definition, Type 1 processes give rise to intuitions [35, 37, 38]. Regarding Type 2 processing, responses are intervened by distinctive higher-order reasoning processes, as they rely heavily on hypothetical thinking and working memory. This type of response is slow, sequential, and correlated with measures of general intelligence [33, 35, 36].

In a recent investigation, participants were exposed to messages without conscious guidance or intention to rely on their emotions or messages to rely on their reasoning to determine if it promotes intentions of using a face-covering mask [2]. The dual-process theories suggest that relying on emotions is typically related to Type 1 processing, whereas relying on reasoning is typically associated with Type 2 processing [36]. Altogether, the three studies conducted revealed that: priming participants with the message to rely on their reasoning increases the choice to wear a face-covering comparatively to priming participants to rely on their emotions [2].

Additionally, researchers have used other techniques like time constraints, ego depletion, and cognitive load besides conceptual priming to activate Type 1 and Type 2 (for a review, see [32]). One of these methods could be more effective at promoting face masks than conceptual priming. Hence, previous research suggests testing which cognitive manipulations best promote face masks [2].

Previous research regarding the cognitive mechanisms involved in cooperation through economic games manipulated cognitive processes, using priming and time constraints methods, suggest that the intuitive response is to cooperate [39–44]. The reported effects lead to the Social Heuristics Hypothesis (SHH) [41, 45]. The SHH assumes that typically successful strategies become automatized as default responses over time. Individuals who generally interact in environments where cooperation is advantageous should be predisposed to cooperation even when it does not pay off [42, 43, 46, 47].

### The present study

Given previous suggestions to test which cognitive manipulations best promote prosocial behaviors [2], the present study aims to empirically investigate the cognitive mechanisms underlying human cooperation by analyzing the impact of intuition on individual prosocial intention under uncertain situations caused by COVID-19. To the best of our knowledge, this study is the first to analyze the interaction between time pressure manipulation and prosocial intentions under a real case of uncertainty caused by a worldwide pandemic.

Consistent with previous SHH findings [43] and the results found in a hypothetical infectious disease scenario [1], we first hypothesize that the prosocial intention will be higher under time pressure conditions than the condition without a time limit. In our second hypothesis, we expected that in decisions concerning the threat of COVID-19, the prosocial intention would be lower in the control condition than in the other two conditions, namely under impact uncertainty and in the worst-case scenario. Moreover, in our third hypothesis, we expect lower prosocial intention under an indirect impact scenario than in the control condition.

Furthermore, we will explore how individual differences influence the decision to adopt protective measures during the COVID-19 pandemic. Our findings will contribute to understanding the determinants of prosocial behaviors and individual differences in health situations. Moreover, this study may provide important insights for encouraging individuals to adopt prosocial behaviors.

## Method

### Participants

A total of 711 participants filled out the questionnaire for this study. However, 215 participants were eliminated from this initial sample as they did not complete the first main task. Therefore, our final sample consisted of 496 subjects (detailed description of the sociodemographic and COVID-19 variables are presented in S1 Table).

Data collection occurred in two different periods of 2020. The first phase consisted of 317 (63.9%) participants who responded to the questionnaire from April until May, and 179 (36.1%) participants responded to the questionnaire in the second phase from October until November. Of the 496 participants, 122 (24.6%) were males and 374 (75.4%) were females, ranging in age from 18 to 61 years old (*M* = 26.11; *SD* = 9.31). The majority were Portuguese (*n* = 483; 97,4%), students (*n* = 262; 52.8%), and most participants (*n* = 209; 58.5%) report having finish the 12^th^ grade. Regarding socioeconomic status (SES), most participants (*n* = 324; 65.3%) report having a medium socioeconomic status.

Most participants (*n* = 407; 81.8%) reported not having interrupted academic or work activity totally because of the pandemic and maintained their activity by working from home or online school (*n* = 309; 62, 9%).

Concerning COVID-19 variables, most participants (n = 283; 47.7%) report not doing quarantine or prophylactic isolation. Participants reported having left home on average 13 times since the begging of the prophylactic isolation. The reasons for leaving home corresponded to the government’s exceptions in the state of emergency (e.g., going to the grocery store; pharmacy). Regarding the infection with the virus at the moment of the questionnaire, the vast majority (*n* = 485; 81.8%) reported not being infected, and most participants (n = 385; 77.6%) reported not having any family or friends infected with COVID-19. All participants gave their written informed consent, according to the Helsinki Declaration. The Ethics Committee for Research in Social and Human Sciences (CEICSH), of the University of Minho, approved the study. There were no exclusion criteria. Participants were naive concerning the whole experimental procedure.

None of the participants received any monetary compensation, and they were recruited through institutional email communication and online social networks (e.g., Facebook).

### Materials

#### Sociodemographic variables questionnaire (Q-SV)

Participants answered several demographic questions regarding their age, sex, nationality, profession, academic qualifications, and socioeconomic status (SES).

#### COVID-19 questionnaire

Regarding COVID-19 questions, participants answered if, due to the COVID-19 pandemic, they completely interrupted their academic or work activity. Suppose participants answered no to the previous question. In that case, they have to clarify if they stayed at home (e.g., working/studying) or if they did not alter their working routine. In addition, participants indicated: if they were or have been infected with the Coronavirus; if any of their family/friends were infected with COVID-19; if they were doing prophylactic isolation or quarantine; how many times they had left home during prophylactic isolation/quarantine; what security and social measures they took during that period. Lastly, participants rate to what extent they believe that in the future, people would comply with the recommendations of the general health direction (such as social distancing, staying at home) on a scale ranging from 1 to 7, *"very unlikely"* (1) to "*very likely*" (7). **Interpersonal Reactivity Index** (IRI; [48, 49]): This scale measures empathy and contains 24 statements about feelings and thoughts that the person may or may not have experienced. The instrument is divided into four subscales (each with six items), namely Perspective Taking (PT), Empathic Concern (EC), Personal Discomfort (PD), and Fantasy (F). Participants were asked to indicate how well each item described them on a 5-point Likert scale, from 1 to 5- "*It does not describe me well*" (1) to "*Describes me extremely well*" (5). The validation to Portuguese confirmed the adequate internal consistency and good reliability of this instrument in the assessment of empathy [49] (PT- Cronbach’s α = 0.73; EC—Cronbach’s α = 0.76; PD—Cronbach’s α = 0.80; F—Cronbach’s α = 0.84).

#### Probabilistic discounting task

Participants answered a probabilistic discounting task based on previous research [50]. Probabilistic discounting (PD) refers to decreasing the subjective value of a reward due to reducing probability. Five probabilities, expressed as percentages (90%, 70%, 50%, 30%, and 10%), were presented in 10 increments of guaranteed money. This task consisted of 45 questions that appeared in a randomized order:

"*Please choose which amount of money you would rather have for each line*:

A. *$75 guaranteed* or B. *A [p]% chance of winning $75*.

A. *$70 guaranteed or* B. *A[p] %chance of winning $75*.---Down To---A. *$5 guaranteed* or B. *A[p]%chance of winning $75*."

#### COVID-19 risk perception scale

To evaluate COVID-19 risk perception, we applied the ten questions used in a previous study [14]. Those questions measured the risk perception regarding infection likelihood and severity for multiple hypothetical ’average’ people and the subject themselves (for example, the average person in the neighborhood, state, and country). Additionally, participants rate the perceived risk of transmitting the infection to another person and how badly that person would be affected. Finally, all items were rated using a visual analog scale ranging from 1 to 7, *"very unlikely"* (1) to "*very likely*" (7). We also questioned people about how badly they felt they had been personally affected by the pandemic on a 7-point Likert scale—*"Not badly"* (1) to "*Badly*" (7).

#### Social Value Orientation (SVO; [51])

The paper-based SVO measure assesses social behavior. In this version, we used the six primary items. First, participants indicate the allocation of points (imagined as money) that defines their most preferred joint distribution between themselves and another person (via a forced choice of nine alternatives that vary benefits to self vs. the other). Then, for the six items, mean allocations for self and the other were calculated. The inverse tangent of these two means’ ratios then produced an angle that indicated the participant’s SVO index. A greater SVO angle suggests that the participant more often chose the option that maximized the other person’s allocation, consistent with prosocial or altruistic behavior.

### Task

Similar to a previous study [1], in the main task of this experiment, participants judged whether a given event was possible or not. To confirm participants internalized the required responses for possible (pressing for the *"possible*" word) versus impossible (pressing for the *"impossible*" word) judgments, they went through a training phase of 20 trials. The words "possible" and "impossible" appeared ten times in random order during the trial practice, and participants had to press the adequate key. Thus, to investigate differences in deliberative forms of modal cognition, we began by focusing specifically on judgments of possibility. To do that, we asked participants to judge if two events were possible or impossible while manipulating the amount of available time for this judgment. These judgments were made based on one of the four scenarios presented to each participant:

#### Control

In the control scenario, participants were presented with the following situation:

"*Considering the Coronavirus (SARS-CoV-2—COVID-19) pandemic health situation*, *you can either be healthy or infected*, *with or without symptoms*. *The General Health Direction has warned that COVID-19 is very contagious*, *meaning that people you contact may get infected*.

*However*, *you still feel able to work*, *and you really want to go to the office to finish a project that is important for your career*".

#### Impact uncertainty

Participants read a similar scenario to the control scenario in the impact uncertainty scenario. However, additional information was added between the previous paragraphs about the vulnerability of people they might infect at their workplace:

"*If you go to your workplace*, *you may infect healthy co-workers for whom COVID-19 is unproblematic and will not develop symptoms*. *However*, *you may also infect colleagues in a more vulnerable risk group for whom the Coronavirus is very dangerous and who may suffer greatly*. *For example*, *people with pre-existing chronic diseases*, *such as cardiovascular disease*, *diabetes*, *or a chronic respiratory disease*.".

#### Worst-case

Participants read a similar scenario to the control scenario in the worst-case scenario. However, additional information was added between the previous paragraphs stating that they would infect a co-worker for whom the infectious disease would be dangerous if they went to their workplace.

"*If you go to your workplace*, *you can infect co-workers who are part of a more vulnerable group*, *for whom the Coronavirus is very dangerous*, *and they might suffer greatly*. *For example*, *people with pre-existing chronic diseases*, *such as cardiovascular disease*, *diabetes*, *or a chronic respiratory disease*.".

#### Indirect transmission

Participants read a similar scenario to the control scenario in the indirect transmission. However, additional information was added between the previous paragraphs suggesting that they could infect somebody for whom the disease would be unproblematic, even though this person could infect people who were part of a more vulnerable group.

"*If you go to work*, *you may infect healthy co-workers for whom COVID-19 is unproblematic and will not develop symptoms*. *However*, *these colleagues may infect people who make part of a more vulnerable group*, *for whom the Coronavirus is very dangerous and who may suffer greatly*. *Such as people with pre-existing chronic diseases like cardiovascular disease*, *diabetes*, *or with a chronic respiratory disease*.".

After reading the scenario presented, participants made two possible judgments (that is, the possibility that co-workers are vulnerable and the possibility of infecting co-workers) by pressing a key to indicate whether they thought the event was possible or impossible. The two possibility judgments were presented in random order. Thus, participants were either forced to make these judgments very quickly (≤ 2 s) or asked to deliberate on the possibility of each event.

Finally, following their possibility judgment, participants proceeded to indicate whether or not they would go to work in the scenario they had read, on a seven-point Likert scale from ’*definitely not’* to ’*definitely’*, also under time pressure (≤ 6 s) or without time limit (> 6 s).

### Procedure

The online questionnaire was developed in Qualtrics software (2020). Participants were randomly assigned to one condition. More specifically, participants were divided into eight conditions in a 4 (COVID-19 scenario: Control vs. Impact Uncertainty vs. Worst-Case vs. Indirect Transmission) × 2 (decision time: time delay vs. time constraint) between-subject design. Participants started by responding to the first two questionnaires regarding sociodemographic variables and questions relative to COVID-19. Then participants answered the main task and started by practicing how to respond to the possibility judgments. After the practice trial, participants read their scenario and receive instructions regarding their experimental condition–time delay or time pressure (similar to [39]). Participants were asked to make each decision as quickly as possible in the time pressure condition. They were told they could not take longer than 2 seconds (in the possibility judgments) and 6 seconds (in the prosocial behavior question of whether they would leave home to go to work). In this condition, a timer appears on the screen to show the time left to decide participants could not respond after the time had ended. On the other hand, in the time delay condition, participants were asked to consider their decision carefully; no timer appeared, and they could choose at any moment. Later, participants responded to the two possibility judgments and the prosocial behavior question of whether they would leave home to go to work in the scenario they had read.

Participants ended the procedure by responding to the four questionnaires evaluating empathy, social value orientation, probabilistic discounting task, and the COVID-19 risk perception scale presented in random order. Participants took, on average, 15 min to complete the questionnaire.

## Data analysis

We first assess the effects of the uncertainty scenarios presented and time manipulation on participants’ likelihood of leaving home by conducting an ordinal logistic regression (OLR) while controlling for the response phase. We expected to observe if the likelihood of leaving home would be higher for the deliberate (no time limit) vs. intuitive (time limit) condition and if this likelihood would change for the different scenarios compared to the control condition. Before analyzing the effects of the individual differences, we extracted the most relevant information from the COVID-19 Risk Perception Scale by performing a principal component analysis (PCA) and a confirmatory factor analysis (CFA). Then, in order to explore whether individual differences could explain the likelihood of leaving home, a second OLR was conducted. More specifically, to analyze if specific variables were predictors of the prosocial decision made, namely: (i) probabilistic discounting task; (ii) the COVID-19 risk perception scale; (iii) question regarding how participants believe in the future people will follow the recommendations of the general health direction; iv) empathy (interpersonal reactivity index); and v) sociodemographic variables: age, gender, and socioeconomic status. Additionally, we conducted ANOVA and Kruskal Wallis test to explore individual differences across the conditions.

Finally, we also performed a multiple linear regression model to understand the contribution of each of the items from the COVID-19 Risk Perception Scale towards the intention of leaving home.

Data analysis was performed using RStudio Version 1.4.1103 [52].

## Results

**Fig 1:**
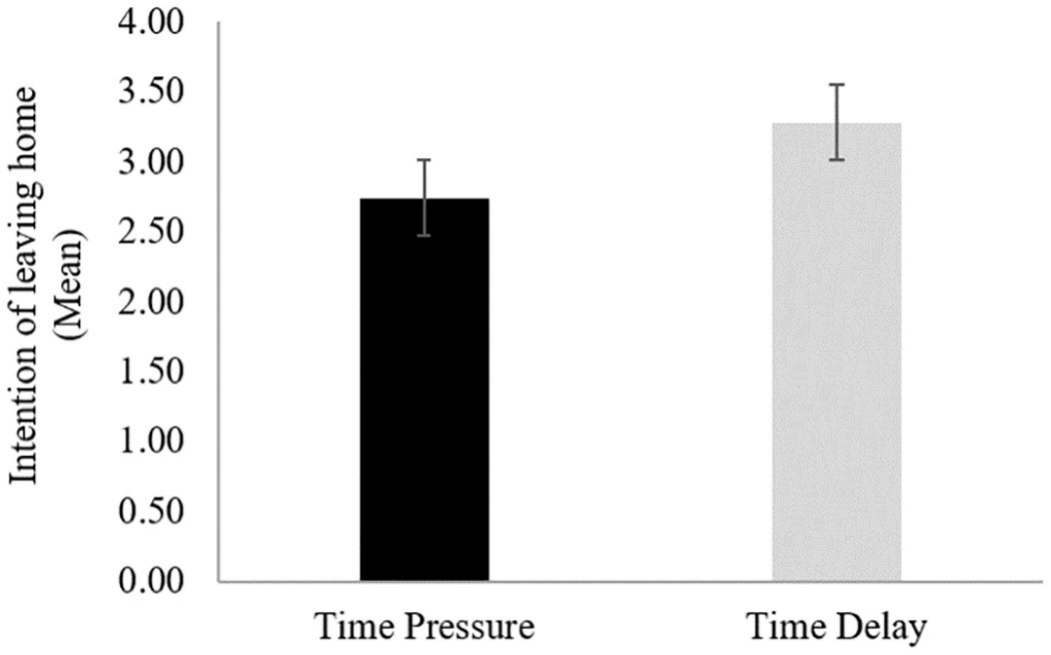
The intention of leaving home for the two types of dual-process manipulation. Error bars represent the standard error of the mean.

Before proceeding with a formal statistical model, we first investigated the data at its mean level. Fig 1 shows the mean decision of leaving home (participants indicated whether or not they would go to work in the scenario presented on a seven-point Likert scale from 1 ’*definitely not’* to 7 ’*definitely*’) in each of the two conditions (time pressure vs. time delay). Regarding the dual-process manipulation, participants in the time pressure condition appeared more likely to stay at home (*M* = 2.74) than the participants without a time limit (*M* = 3.28).

**Fig 2:**
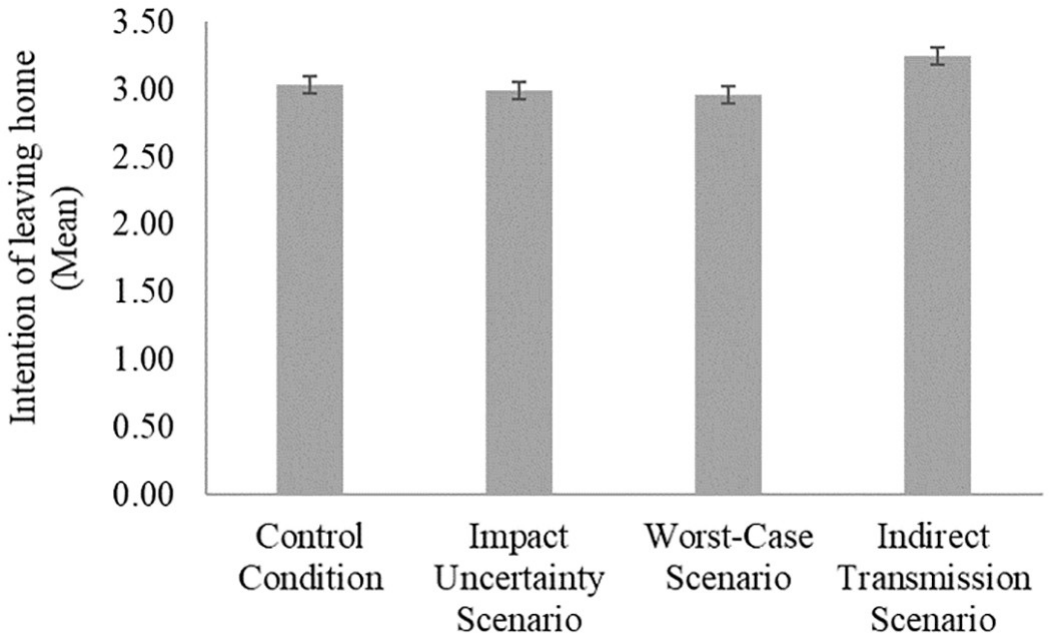
The intention of leaving home for the four scenarios presented. Error bars represent the standard error of the mean.

Regarding the uncertainty manipulation conditions, mean data reveal that participants in the impact uncertainty and worst-case conditions have a lower mean decision to leave home when compared to the median decision of participants in the control condition. Conversely, participants in the indirect transmission scenario have a higher mean intention to leave home when compared to the mean data of the other three scenarios (see Fig 2). In order to confirm these results, an ordinal regression was performed.

The first OLR was composed of both time pressure and scenario main effects entered as predictors and the intention to leave home to work as the dependent variable while controlling for the response phase (see Table 1). The model revealed that time pressure had a statistically negative effect on the likelihood of leaving home (*β* = -.50, *SE* = 0.16, *t*(494) = -3.09, *p* = .002, *OR* = 0.61, 95% *CI* = 0.44, 0.83). The odds ratio was 0.61, which means that for the time limit condition, the probability of leaving home was 0.60 lower than for the no time limit.

**Table 1:** Estimates of the ordinal logistic regression model.

	*β (SE)*	*t value*	*p*	95% CI for odds ratio		
				Lower	Odds Ratio	Upper
Intercept						
Time manipulation	-.50(.16)	-3.09	.002**	.44	.61	.83
Scenario: Impact Uncertainty	-.02(.23)	-.08	.938	.63	.98	1.54
Scenario: Worst-Case	-.00(.22)	-.01	.994	.65	1.00	1.54
Scenario: Indirect Transmission	.19(.23)	.84	.400	.77	1.21	1.90
Response Phase	.20(.17)	1.18	.238	.88	1.22	1.69
AIC	888.34					
Number of obs.	496					

Note: AIC- The Akaike information criterion; CI- Confidence Interval; SE- Standard Error of the coefficient; β—Coefficient.

*** p < .001,

** p < .01,

* p < .05.

Relatively to the scenarios condition, no differences were found between the different scenarios in comparison to the control scenario condition: impact uncertainty (*β* = -.02, *SE* = 0.23, *t*(244) = -0.08, *p* = .94, *OR* = 0.98, 95% *CI* = 0.63, 1.54); worst-case (*β* = -.002, *SE* = 0.22, *t*(253) = -0.07, *p* = .99, *OR* = 1.54, 95% *CI* = 0.65, 1.54); nor for the indirect transmission (*β* = .19, *SE* = 0.23, *t*(247) = 0.84, *p* = .40, *OR* = 1.21, 95% *CI* = 0.77, 1.90). Also, the response phase did not show a significant effect (*β* = .20, *SE* = 0.17, *t*(494) = 1.18, *p* = .24, *OR* = 1.22, 95% *CI* = 0.88, 1.69).

### Dimensionality reduction from the COVID-19 risk perception scale

An initial principal component analysis with varimax rotation indicated that three dimensions should be extracted from the COVID-19 Risk Perception Scale: perceived spread (3 items), perceived impact (4 items), and perceived distant spread (2 items). Thus, confirmatory factor analysis was performed, which showed a good fit (χ^2^ = 57.99, *df* = 24, *p* < .001; comparative fit index (CFI) = .94; Tucker–Lewis index (TLI) = .92;root mean square error of approximation (RMSEA) = .07. The relative figure and loadings from the confirmatory factor analysis are presented in S1 Fig and S2 Table.

### Ordinal logistic regression (OLR) for the individual differences

The second OLR was composed of the participant’s response to the: probabilistic discounting task; the COVID-19 risk perception scale; the question regarding how participants believe in the future people will follow the recommendations of the general health direction; the interpersonal reactivity index; age; gender; and socioeconomic status.

The model was comprised of only 254 entries due to not all participants having completed the full questionnaire. Table 2 presents the results found in the ordinal logistic regression for the individual differences. We found a negative significant effect for the perceived COVID-19 impact (*β* = -.21, *SE* = 0.11, *t*(252) = -2.03, *p* = .03, *OR* = 0.81, 95% *CI* = 0.66, 0.99), a negative effect of age (*β* = -.04, *SE* = 0.02, *t*(252) = -2.13, *p* = .03, *OR* = 0.96, 95% *CI* = 0.92, 1.00), and a significant negative effect between subjects with higher socioeconomic levels compared to individuals with lower socioeconomic conditions (*β* = -.78, *SE* = 0.38, *t*(87) = -2.02, *p* = .04, *OR* = 0.46, 95% *CI* = 0.22, 0.98), and an effect approaching the significance level for individuals with moderate socioeconomic levels (*β* = -.51, *SE* = 0.29, *t*(214) = -1.76, *p* = .08, *OR* = 0.60, 95% *CI* = 0.34, 1.06).

**Table 2:** Estimates of the ordinal logistic regression model for the individual differences.

	*β (SE)*	*t value*	*p*	95% CI for odds ratio		
				Lower	Odds Ratio	Upper
Intercept						
Probabilistic Discounting Task: AUC	.90(.54)	1.67	.10	.85	2.47	7.11
COVID-19 Risk Perception Scale: Perceived Spread	.17(.11)	1.66	.10	.97	1.91	1.46
COVID-19 Risk Perception: Perceived Distant spread	-.05(.15)	.33	.74	.79	1.05	1.40
COVID-19 Risk Perception: Perceived Impact	.-.21(.11)	-2.03	.04*	.66	.81	.99
IRI-Perspective Taking	.05(.03)	1.52	.13	.99	1.05	1.25
IRI-Empathic Concern	-.01(.05)	-.24	.81	.95	.99	1.00
IRI-Personal Distress	-.05(.03)	-1.83	.07	.89	.95	1.00
IRI-Fantasy	-.00(.03)	-.13	.90	.95	1.00	1.05
Gender	.41(.37)	1.10	.27	.72	1.50	3.13
Age	-.04(.02)	-1.83	.03*	.92	.96	1.00
Socioeconomic Status: level 2	-.51(.29)	-1.76	.08	.34	.60	1.06
Socioeconomic Status: level 3	.77(.38)	-2.02	.04*	.22	.46	1.00
Follow the recommendations of the general health direction	-.06(.10)	-.64	.52	.66	.81	.99
AIC	888.34					
Number of obs.	254					

Note: AIC- The Akaike information criterion; CI- Confidence Interval; SE- Standard Error of the coefficient; β—Coefficient.

*** p < .001,

** p < .01,

* p < .05.

The other individual variables present in the model did not reveal to be significant predictors of the intention of leaving home (see Table 2).

Additionally, we conducted ANOVA and Kruskal-Wallis tests and found no effect on individual differences across the different conditions, except for a significant effect in the socioeconomic status (χ2 = 15.34, *p* = .03) (see S3 Table). However, pairwise post-hoc Dunn test with Bonferroni adjustments did not reveal differences in the socioeconomic status across the conditions (see S4 Table).

### The predictive power of the COVID-19 risk perception scale

**Fig 3:**
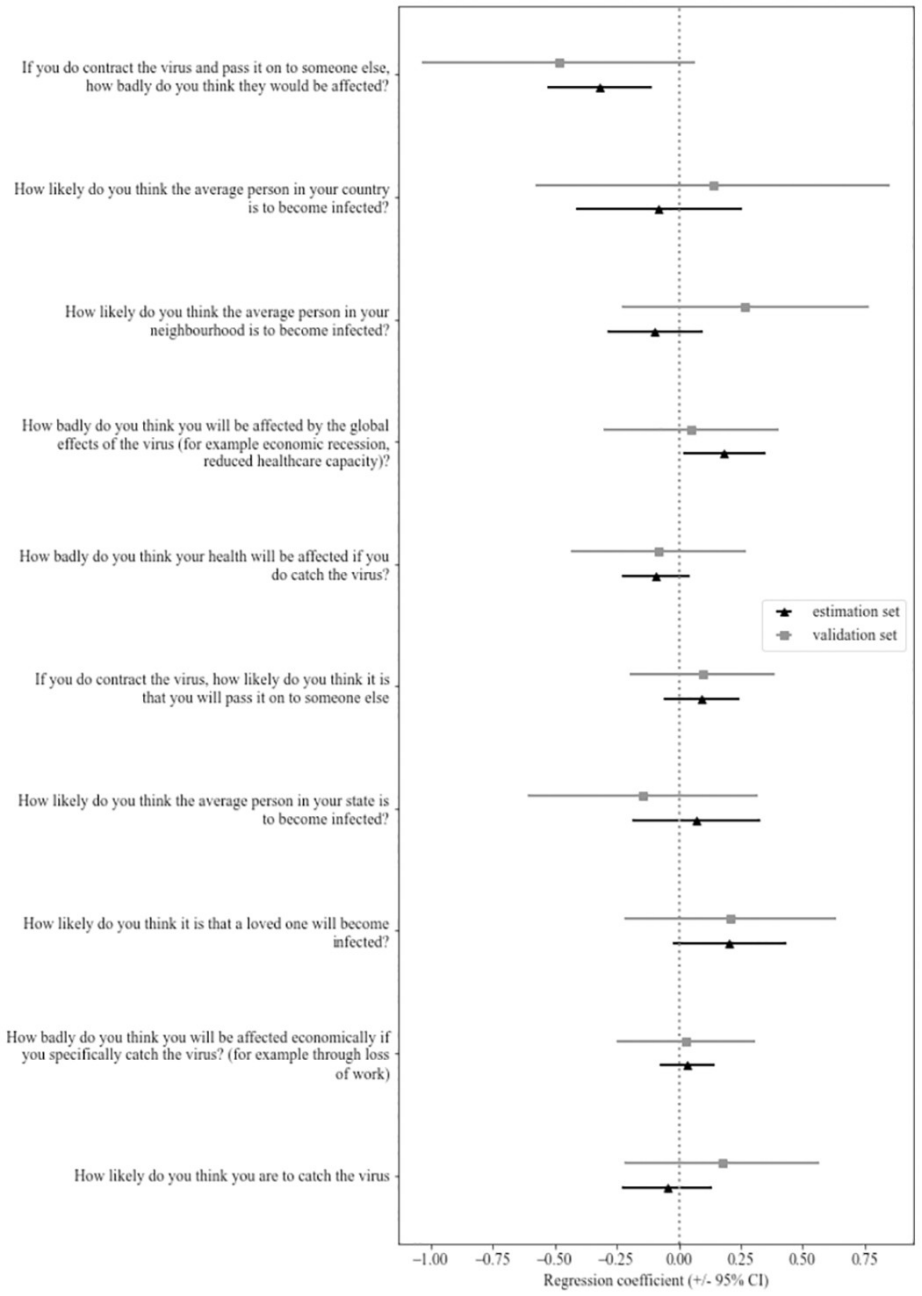
Results of linear regression predicting the intention of leaving home from risk perception measures, with validation in a subsample of 20% of the subjects.

To understand the contribution of each of the ten items from the COVID-19 risk perception scale towards the intention of leaving home, we performed a multiple linear regression model. To ensure reproducibility of our results, we performed this analysis in a subset consisting of 80% of participants and repeated it in a validation set with the remaining 20%. The results reported here are from the complete database, although they are consistent with findings in both subsets. We want to highlight that the perceived negative effects for another individual that the participant infected decreased the intention of leaving home (*β* = -.39, *t*(262) = -3.96, *p* < .001). Surprisingly, none of the other items showed a significant effect (see Fig 3).

## Discussion

The main objective of the present study was to analyze the impact of intuition on individual prosocial intention under uncertainty caused by COVID-19. For that, we manipulated the uncertainty conditions based on fictive scenarios set in the context of COVID-19. In the control condition, participants read about the situation caused by COVID-19, its spread, and how people they contact may get infected. Across the uncertainty conditions, we vary the information regarding the vulnerability of those they might infect. After reading the scenario presented, participants indicated whether or not they would leave home to go to work in the scenario they had read, either under time pressure (intuition) or without a time limit (deliberation).

### Effects of intuition on prosocial behavior

Consistent with our first hypothesis, our results revealed that participants in a time pressure condition diminished their intentions of leaving home to go to work relative to participants without a time limit. These findings suggest that intuition favors prosocial intention. Additionally, our results support previous research that observes that participants who made implicit possibility judgments (under time pressure) were less likely to indicate they would go to work—prosocial behavior—than those who made explicit possibility judgments (under a time delay) [1]. Additionally, previous research has demonstrated that time pressure manipulation increases cooperative behavior in experimental economic games [39, 43, 44]. These findings contribute to the study of the SHH by expanding the knowledge in this area to real-case scenarios and not only to economic games. Considering the SHH, people adopt approaches that are generally advantageous and effective in their social interactions when their capacity to deliberate is impaired [32, 43, 44]. Social norms and values regulate how individuals live in communities [53, 54]. Social enforcement mechanisms encourage people to embrace and internalize shared norms, motivating them to do what is considered correct while avoiding behaviors that seem wrong [55]. In the COVID-19 uncertainty scenario presented, participants have internalized prosocial behavior, which means that individuals internalize the preventive measures of staying at home recommended by the local governments and health authorities.

Recent research suggested that it is essential to analyze which cognitive manipulations best promote the acceptance of preventive behaviors [2]. The authors use conceptual primes of emotion and reason to activate Type 1 and Type 2 cognitive functioning in their study. Results reveal that priming reasoning significantly increases intentions to wear a face-covering mask than priming people to rely on their emotions. However, our results suggest that time pressure increases the prosocial decision to stay at home. Considering these two results, future research using the same procedure should explore the cognitive basis of prosocial behavior during COVID-19 using different procedures like conceptual primes, ego-depletion, cognitive load, two-response paradigm, and neurostimulation [32].

### Effects of uncertainty on prosocial behavior

Our data did not confirm our second and third hypotheses regarding the uncertainty’s effect on cooperation. Our second hypothesis expected prosocial intention to be higher under impact uncertainty and in the worst-case scenario than the control condition. Moreover, in the third hypothesis, we expected lower prosocial intention under an indirect impact scenario than in the control condition. However, our results reveal no difference across the different scenarios read in the decision to leave home. As in previous studies, we found out that uncertainty does not always decrease prosocial behavior [1]. However, we do not find the same pattern. Our results show that the type of uncertainty did not have an effect on the prosocial intention when participants answered to an actual infectious disease case.

Moral decision-making typically involves uncertainty during a pandemic once it is uncertain whether one is infected and will infect others [9]. People may be less willing to cooperate with others when the benefits are uncertain [1, 30]. However, in the previous research, focusing on worst-case scenarios may have encouraged people to make sacrifices for others even if the benefits were uncertain [1]. In addition, when people make moral decisions, they often consider how others would judge them for behaving selfishly [9]. Our results suggest that moral decisions are of great importance in a pandemic crisis, implying that participants want to maintain a good reputation by staying home and complying with health authorities’ orientations. Since disrespecting the preventive measures and social norms in the pandemic will be severely judged.

We can also consider that we did not find an effect since our baseline condition was too close to the uncertainty we are currently living. In the control condition, participants were asked to consider the possibility of being infected and asymptomatic or not infected. Since this condition presents uncertainty, it may not be the best control condition, and future research should compare the uncertainty conditions with no exposure. Possibly, the awareness of infection rates and the likelihood of severe symptoms caused by COVID-19 reduces all conditions to the impact uncertainty condition.

### Effects of the individual differences on prosocial behavior

Considering previous research on how individual differences may influence cooperative and prosocial behavior [18, 19, 56, 57], we explore how individual differences (probabilistic discounting, COVID-19 risk perception, empathy, age, gender, SES) predicts prosocial behavior during the COVID-19 pandemic.

Results revealed that older individuals report a lower intention to leave home. This result is consistent with the literature since older individuals have a higher risk of suffering badly from COVID-19, so the perceived risk for these individuals is higher than the young adults [5, 58]. Additionally, we found that socioeconomic status is a predictor of prosocial behavior. The results indicate that participants who report a medium-high socioeconomic status have less intention of leaving home than participants who report having a medium-low socioeconomic status. Previous research reveals that socioeconomic characteristics do not affect cooperative behavior [20]. However, the socioeconomic variables influence trust attitudes, which are correlated with people’s contribution behavior [20]. In particular, people’s socioeconomic status has a significant impact on their trust in the fairness of others and their fear of being exploited by others. In other words, those who believe that others will not exploit them cooperate more than those who believe the opposite [20].

We also found out that the perceived COVID-19 impact is a significant predictor of the intention of leaving home. When participants perceived a higher rate of negative impact from the global, economic, and health consequences of COVID-19, they were more likely to leave home. Thus, special attention should be given to the communication and nudging of more vulnerable groups (e.g., people with poorer socioeconomic status) to adopt preventive measures since these groups generally suffered more during the pandemic.

The multiple linear regression results revealed that participants’ intention of leaving home decreases if they infected someone, and the person would suffer badly. This effect is consistent with our results and previous findings since focusing on how badly others could suffer from the disease will increase prosocial behavior [1].

The other individual differences do not reveal a significant effect in predicting the intention of leaving home. In our analysis, we decided not to apply the social value orientation since our sample was too homogeneous. More specifically, most of our participants were prosocially oriented individuals (88%). However, a recent study found no evidence that SVO influenced either the perception or the frequency of protective measures [58]. Like our sample, most participants in their study were categorized as prosocial [58].

Regarding the limitations of our study, the first is that our sample was relatively young, with a median age (26.11 years), and mainly consisted of women (75.4%). Our sample was at low risk of serious illness due to the pandemic, primarily female and young [5, 58]. Therefore, the observed optimism bias could be based on being genuinely lower risk than the average person. Another limitation in our study that could influence participants’ decision to stay home is that most of our sample were university students for whom the possibility of working from home was not problematic. So, our scenario manipulation could not have the impact we would imagine.

## Conclusion

Several studies have analyzed different strategies for engaging in effective science communication, persuasion around public health, and nudging people to adopt preventive measures [2, 10, 11]. These studies have focused on preventive measures like face-covering masks and social distancing [2, 10]. On the other hand, our study focuses on whether participants decide to leave home to go to work or not since the lockdown measure is one of the most restrictive preventive measures the local governments have applied.

With this study, we contribute to the literature by revealing that pparticipants were more inclined to behave in a more prosocial manner when forced to make their decision quickly rather than deliberately on actual infectious disease. Our results showed that time pressure manipulation significantly impacts prevention intention even after preventive behaviors have become a widespread social concern. Additionally, our findings revealed that the uncertainty presented does not matter when presented with an actual infectious disease, and participants adopt preventive behaviors anyway.

Even though most of the countries soften the restrictive measures as the vaccination proceeds, the fight against COVID-19 is still uncertain since it will depend on the duration of vaccine protection, emergence or not of new variants, and people’s behavior. Therefore, messages that focus on the uncertainty of people contracting and spreading the disease, urgency appeals, and intuition priming can help to keep the public on its guard against the disease and promote preventive behaviors within the population.

## Supporting information

S1 Table Description of the sociodemographic and COVID-19 variables. Results are shown in absolute and relative (%) frequencies.
(DOCX)

S2 Table Confirmatory factor analysis. Standardized loadings for the COVID-19 Risk Perception Scale items.
(DOCX)

S3 Table ANOVA and Kruskal-Wallis tests for the individual difference’s variables across the different conditions. (DOCX)

S4 Table Pairwise post-hoc Dunn test with Bonferroni adjustments. Results from the different conditions on the socioeconomic status.
(DOCX)

S1 Fig Three-factor model of the COVID-19 risk perception scale. (TIF)

S1 File Database. (XLSX)
