# Supplementary material for: A dual-process approach to prosocial behavior under COVID-19 uncertainty
Source: PLoS One. 2022 Mar 29;17(3):e0266050. doi: 10.1371/journal.pone.0266050 (PMC8963555; doi:10.1371/journal.pone.0266050)
Supplement: S1 Table — Results are shown in absolute and relative (%) frequencies. (DOCX) [file pone.0266050.s001.docx]

**S1 Table. Description of the sociodemographic and COVID-19 variables.** Results are shown in absolute and relative (%) frequencies

| Personal characteristics | Level | n (%) |
| --- | --- | --- |
| Sex | Male | 122 (24.6) |
|  | Female | 374 (75.4) |
| Nationality | Portuguese | 483 (97.4) |
|  | Brazilian | 10 (2.0) |
|  | Venezuelan | 1 (0.2) |
|  | Sao Tomean | 1 (0.2) |
|  | Spanish | 1 (0.2) |
| Highest academic qualification | 6^th^ Grade | 2 (0.4) |
|  | 9^th^ Grade | 15 (3.0) |
|  | 12^th^ Grade | 290 (58.5) |
|  | Bachelor of Arts/Science | 106 (21.4) |
|  | Master | 77 (15.5) |
|  | Postgraduate | 2 (0.4) |
|  | Doctorate | 4 (0.7) |
| Socio-economic status | Low | 14 (2.8) |
|  | Medium-low | 103(20.8) |
|  | Medium | 324 (65.3) |
|  | Medium-high | 55 (11.1) |
| Employment status | Employed | 161 (32.5) |
|  | Student | 262 (52.8) |
|  | Working student | 46 (9.3) |
|  | Unemployed | 15 (3.0) |
|  | Did not reveal | 12 (2.4) |
| COVID-19 work situation | Remote | 309 (62.3) |
|  | On-site | 97 (16.4) |
|  | Stay home without working | 39 (6.6) |
|  | B-learning | 8 (1.3) |
|  | Work in fortnightly periods | 7 (1.2) |
|  | Informal Caregiver | 2 (0.3) |
|  | Student Worker | 30 (5.1) |
|  | Did not answer | 4 (0.7) |
| Doing quarantine or prophylactic isolation | Yes | 213 (35,9) |
|  | No | 283 (47,7) |
| COVID-19 infection | Infected at the moment | 2 (0,3) |
|  | Already been infected | 4 (0.7) |
|  | Not being infected | 485 (81.8) |
|  | Do not know | 5 (0.8) |
| Family/Friends COVID-19 infection | Had friends/family infected | 35 (7.1) |
|  | Have friends/family already been infected | 76 (15.3) |
|  | Not have friends/family infected | 385 (77,6) |
