# Supplementary material for: A dual-process approach to prosocial behavior under COVID-19 uncertainty
Source: PLoS One. 2022 Mar 29;17(3):e0266050. doi: 10.1371/journal.pone.0266050 (PMC8963555; doi:10.1371/journal.pone.0266050)
Supplement: S2 Table — Standardized loadings for the COVID-19 Risk Perception Scale items. (DOCX) [file pone.0266050.s002.docx]

**S2 Table. Confirmatory factor analysis standardized loadings for the Subjective Covid-19 Risk Perception Scale items.**

| Item number | Item | Perceived spread of COVID-19 | Perceived impact of COVID-19 | Perceived distant spread of COVID-19 |
| --- | --- | --- | --- | --- |
| 1 | How likely do you think you are to catch the virus | 0.72 |  |  |
| 2 | How badly do you think you will be affected economically if you specifically catch the virus? (for example through loss of work) |  | 0.48 |  |
| 3 | How likely do you think it is that a loved one will become infected? | 0.88 |  |  |
| 4 | How likely do you think the average person in your state is to become infected? |  |  | 0.99 |
| 5 | How badly do you think your health will be affected if you do catch the virus? |  | 0.70 |  |
| 6 | How badly do you think you will be affected by the global effects of the virus (for example economic recession, reduced healthcare capacity)? |  | 0.43 |  |
| 7 | How likely do you think the average person in your neighbourhood is to become infected? | 0.61 |  |  |
| 8 | How likely do you think the average person in your country is to become infected? |  |  | 0.49 |
| 9 | If you do contract the virus and pass it on to someone else, how badly do you think they would be affected? |  | 0.63 |  |
