# Supplementary material for: A dual-process approach to prosocial behavior under COVID-19 uncertainty
Source: PLoS One. 2022 Mar 29;17(3):e0266050. doi: 10.1371/journal.pone.0266050 (PMC8963555; doi:10.1371/journal.pone.0266050)
Supplement: S3 Table — (DOCX) [file pone.0266050.s003.docx]

**S3 Table. Results from the ANOVA and Kruskal-Wallis tests for the individual differences variables across the different conditions.**

|  | χ2 | *F* | *p* |
| --- | --- | --- | --- |
| Probabilistic Discounting Task: AUC |  | 0.27 | .85 |
| Subjective Covid-19 Risk Perception: perceived spread |  | 0.56 | .29 |
| Subjective Covid-19 Risk Perception: Perceived distant spread |  | 1.27 | .29 |
| Subjective Covid-19 Risk Perception: Perceived Impact |  | 1.75 | .16 |
| IRI-Perspective Taking |  | 0.80 | .49 |
| IRI-Empathic Concern |  | 0.66 | .58 |
| IRI-Personal Distress |  | 1.65 | .18 |
| IRI-Fantasy |  | 0.98 | .40 |
| SVO | 10.20 |  | .18 |
| Gender | 9.95 |  | .19 |
| Age | 0.54 |  | .66 |
| Socio-Economic Status | 15.34 |  | .03 |
| Follow the recommendations of the general health direction | 13.60 |  | .06 |
