# Supplementary material for: A dual-process approach to prosocial behavior under COVID-19 uncertainty
Source: PLoS One. 2022 Mar 29;17(3):e0266050. doi: 10.1371/journal.pone.0266050 (PMC8963555; doi:10.1371/journal.pone.0266050)
Supplement: S4 Table — Results from the different conditions on the socioeconomic status. (DOCX) [file pone.0266050.s004.docx]

**S4 Table. Results from the pairwise post-hoc Dunn test with Bonferroni adjustments for the socioeconomic status across the different conditions.**

| Comparison | Z | *p adj* |
| --- | --- | --- |
| 1 - 2 | -0.70 | 1.00 |
| 1 - 3 | -1.79 | 1.00 |
| 2 - 3 | -1.04 | 1.00 |
| 1 - 4 | -0.40 | 1.00 |
| 2 - 4 | 0.30 | 1.00 |
| 3 - 4 | 1.37 | 1.00 |
| 1 - 5 | -2.44 | .41 |
| 2 - 5 | -1.78 | 1.00 |
| 3 - 5 | -0.89 | 1.00 |
| 4 - 5 | -2.08 | .91 |
| 1 - 6 | 0.22 | 1.00 |
| 2 - 6 | 0.84 | 1.00 |
| 3 - 6 | 1.80 | 1.00 |
| 4 - 6 | 0.58 | 1.00 |
| 5 - 6 | 2.41 | .41 |
| 1 - 7 | -1.93 | 1.00 |
| 2 - 7 | -1.26 | 1.00 |
| 3 - 7 | -0.33 | 1.00 |
| 4 - 7 | -1.55 | 1.00 |
| 5 - 7 | 0.52 | 1.00 |
| 6 - 7 | -1.94 | 1.00 |
| 1 - 8 | -2.44 | .40 |
| 2 - 8 | -1.78 | 1.00 |
| 3 - 8 | -0.90 | 1.00 |
| 4 - 8 | -2.07 | .89 |
| 5 - 8 | -0.02 | .98 |
| 6 - 8 | -2.41 | .40 |
| 7 - 8 | -0.53 | 1.00 |
