# Supplementary figures and images for: A dual-process approach to prosocial behavior under COVID-19 uncertainty
Source: PLoS One. 2022 Mar 29;17(3):e0266050. doi: 10.1371/journal.pone.0266050 (PMC8963555; doi:10.1371/journal.pone.0266050)

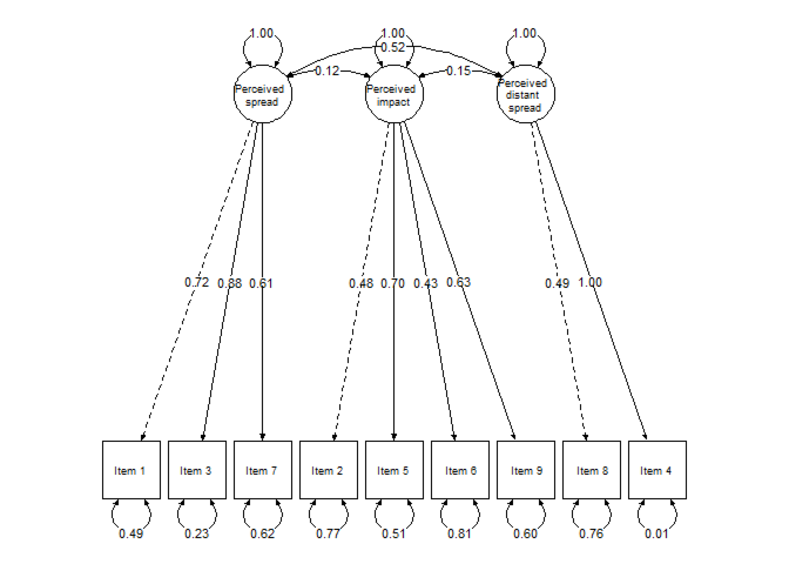

Supplement: S1 Fig — (TIF) [file pone.0266050.s005.tif]
